# Growth comparison of several *Escherichia coli* strains exposed to various concentrations of lactoferrin using linear spline regression

**DOI:** 10.1186/2042-5783-2-5

**Published:** 2012-04-16

**Authors:** Camilla Sekse, Jon Bohlin, Eystein Skjerve, Gerd E Vegarud

**Affiliations:** 1 Norwegian Veterinary Institute, P. O. Box 750 Dep, N-0106, Oslo, Norway; 2 Norwegian School of Veterinary Science, Epi-Centre, Department of Food Safety and Infection Biolog, P.O. Box 8146 Dep, N-0033, Oslo, Norway; 3 Norwegian University of Life Sciences, Department of Chemistry, Biotechnology and Food Science, P.O. Box 5003, N-1432, Aas, Norway

**Keywords:** Escherichia coli, Growth curves, Spline regression

## Abstract

**Background:**

We wanted to compare growth differences between 13 *Escherichia coli* strains exposed to various concentrations of the growth inhibitor lactoferrin in two different types of broth (Syncase and Luria-Bertani (LB)). To carry this out, we present a simple statistical procedure that separates microbial growth curves that are due to natural random perturbations and growth curves that are more likely caused by biological differences.

Bacterial growth was determined using optical density data (OD) recorded for triplicates at 620 nm for 18 hours for each strain. Each resulting growth curve was divided into three equally spaced intervals. We propose a procedure using linear spline regression with two knots to compute the slopes of each interval in the bacterial growth curves. These slopes are subsequently used to estimate a 95% confidence interval based on an appropriate statistical distribution. Slopes outside the confidence interval were considered as significantly different from slopes within. We also demonstrate the use of related, but more advanced methods known collectively as generalized additive models (GAMs) to model growth. In addition to impressive curve fitting capabilities with corresponding confidence intervals, GAM’s allow for the computation of derivatives, *i.e.* growth rate estimation, with respect to each time point.

**Results:**

The results from our proposed procedure agreed well with the observed data. The results indicated that there were substantial growth differences between the *E. coli* strains. Most strains exhibited improved growth in the nutrient rich LB broth compared to Syncase. The inhibiting effect of lactoferrin varied between the different strains. The atypical enteropathogenic aEPEC-2 grew, on average, faster in both broths than the other strains tested while the enteroinvasive strains, EIEC-6 and EIEC-7 grew slower. The enterotoxigenic ETEC-5 strain, exhibited exceptional growth in Syncase broth, but slower growth in LB broth.

**Conclusions:**

Our results do not indicate clear growth differences between pathogroups or pathogenic versus non-pathogenic *E. coli.*

## Background

Randomness is a natural part of biological systems and can make comparisons between biological entities difficult. The challenges lie in separating truly different phenomena from random perturbations. The aim of this study was to compare, with statistical accuracy, the growth of 13 *Escherichia coli* strains subjected to varying concentrations of the growth inhibitor lactoferrin.

*E. coli* is a complex group of bacteria comprising mostly harmless commensals that are normal inhabitants of the intestinal tract of all warm-blooded animals including humans. A subgroup of *E. coli* has been proposed as candidates for probiotic treatment of enteric diseases, while other subsets have acquired different sets of virulence factors that may cause intestinal and extra-intestinal disease. Most pathogenic *E. coli* follow a common strategy for infection based on adhesion and colonization of epithelial cells in the host, evasion of host defenses, multiplication and host damage [1]. Diarrhoeagenic *E. coli* consist of six pathogroups based on different virulence factors, clinical symptoms and serotypes: Enteropathogenic *E. coli* (EPEC), enterotoxigenic *E. coli* (ETEC), enteroinvasive *E. coli* (EIEC), enteroaggregative *E. coli* (EAEC), Shiga toxin-producing *E. coli* (STEC) and diffusely adherent *E. coli* (DAEC) [2].

Lactoferrin is an iron-binding protein in secretions such as milk, tears and saliva, and has antibacterial effect based on two distinct mechanisms [3,4]. Lactoferrin inhibits growth by its ability to bind ferric iron, and limiting this essential nutrient result in bacteriostasis [4,5]. In addition, lactoferrin may prevent bacterial adhesion and invasion of mammalian cells by interfering with surface expressed virulence factors and thereby decrease virulence [4]. Decreased adhesion to epithelial cells caused by lactoferrin has been reported for several *E. coli* pathogroups such as EPEC, STEC, EAEC and ETEC [6-8], and decreased invasion has been identified for EIEC [9].

Antibacterial effect is often studied as minimal inhibitory concentration (MIC) of the tested sample at one time point, usually after bacterial growth over night. Studying growth continuously over time is of importance for observing how and when bacteria respond to an antibacterial substance. However, associating explanatory variables with continuous bacterial growth curves, known as longitudinal studies, using traditional statistical methods such as ordinary least squares regression (OLS) is difficult due to the dependence between the measured points. In addition, bacterial growth, as measured using optical densities, can lead to non-linear curves that are difficult to handle with linear OLS-based methods.

Modeling of bacterial growth is commonly divided into two branches, namely predictive and descriptive models (See [10] and references therein). Examples of the former include mathematical models using largely different types of differential equations [11]. These models are usually supplied by a list of initial conditions that are instrumental in predicting the progress of bacterial growth. Predictive models therefore assume a predetermined rule or causation of how bacterial cultures evolve with time. Descriptive models on the other hand are based on statistical inference, *i.e.* they are descriptive in the sense that no predetermined rule is assumed. Hence, descriptive models can be used to examine the association between a set of relevant external factors with the observed growth trends. Such methods can also be used to analyze and compare differences in and between bacterial growth curves.

Due to the nature of the growth curve data standard statistical models will easily find differences between growth curves; the challenge lies however in separating the truly different growth curves from the similar curves differing only as a result of random fluctuations in growth. In the approach taken here the slopes, or growth rates, are calculated for each bacterial growth curve, which in this study consists of OD measurements with respect to time. Since bacterial growth varies with time, each curve is divided into three intervals of equal duration of which a slope is calculated. The focus here is on comparing the growth of the different strains and not modeling the growth *per se*. For the comparisons to be as accurate as possible each interval must contain the same amount of points so that a slope can be computed from line segments of identical length (*i.e.* duration in our case since the data is time dependent). Since the length of the lag, exponential and stationary phases of the growth curves vary considerably among the different strains, modeling and comparing these phases exactly using the procedure applied here will make the analyses inaccurate and cumbersome. We have therefore opted for a more unusual way of comparing growth curves that focuses on growth change over three equal intervals for each curve. This is referred to as growth rate in the present study, but since growth is measured using absorbance it should be understood as OD rate. We assert that the growth rate of a set of bacterial strains is similar if the calculated slopes are within a 95% confidence interval found using, for instance, a t-distribution. Conversely, we consider slopes, or growth rates, inside the 95% confidence interval to be significantly different from the ones outside. We claim that this approach makes it fast and easy to statistically compare many bacterial growth curves.

There exist several extensions to OLS based methods such as generalized linear regression models (GLM) [12], Gompertz based models and others [13] that can be used to analyze and compare bacterial growth and similar types of studies. A recent approach used a non-parametric mixed effects-model based regression with random effects to handle the dependence between each consecutive point found in growth curve data [10]. The regression model was further improved by bootstrapping the regression coefficients so that a prediction band could be obtained for the modeled growth curve. We also demonstrate the use of GAM regression, which is an extension to spline regression, to model bacterial growth in one of the *E. coli* strains. GAMs are almost as easy to set up as standard linear regression models, but the use of splines makes GAMs better at modeling longitudinal data. Not only can a confidence interval be obtained for the whole curve, but we also show that derivatives, with confidence intervals, of the fitted curves can be easily computed, revealing more information with respect to how growth changes at each time point.

## Results

### The growth curve models

**Figure 1:**
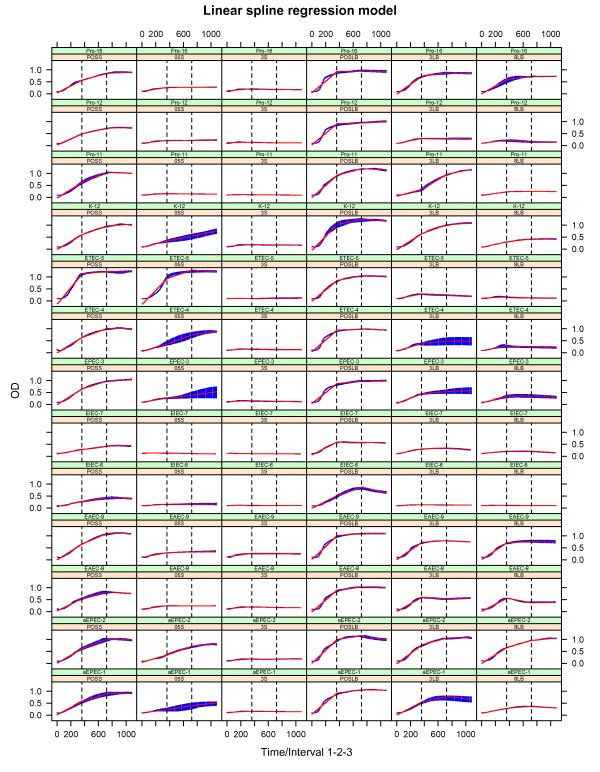
***E.coli*growth curves.** The figure shows the bacterial growth curves for 13 different *E. coli* strains exposed to different amounts of lactoferrin growing in LB and Syncase broth. The horizontal axes represent time in minutes, while the vertical axes show optical density illustrating bacterial growth as measured using OD at 620 nm. For both LB and Syncase broth three different concentrations of lactoferrin were added: 0, 3, 8 mg/ml (designated POSLB, 3LB and 8LB, respectively) for LB and 0, 0.5, 3 mg/ml (designated POSS, 05 S, and 3 S, respectively) for Syncase. The blue line represents the empirical growth curve of the triplicates, while the red line represents the linear spline regression modeled growth curve. The dashed vertical lines represent the knots dividing the spline modeled growth curve into three intervals. The estimated slopes of each line segment resulting from the spline regression were used to compare the growth rates of the different strains.

**Figure 2:**
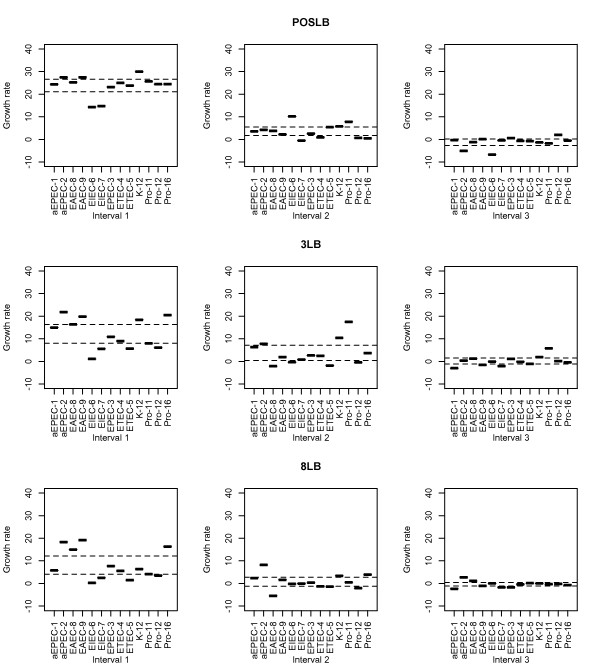
**Comparison of growth rate coefficients in LB broth.** The diagrams depict growth rate coefficients as estimated with the spline regression model between the different strains in interval 1, 2 and 3 cultured in LB broth with 0, 3 and 8 mg/ml (designated POSLB, 3LB, 8LB, respectively) lactoferrin added. The horizontal axes represent the different strains, while the growth rate, measured as change of OD per interval x 1000, is given on the vertical axes. Dashed horizontal lines represent 95% confidence intervals for each interval in each trial (*i.e.* POSLB, 3LB, 8LB). Growth rates, here represented as small bars, above the 95% confidence intervals were regarded as significantly higher compared to the growth rates within or below the dashed lines. Similarly, growth rates below the 95% confidence intervals were regarded as significantly lower than the growth rates within or above the dashed lines.

**Figure 3:**
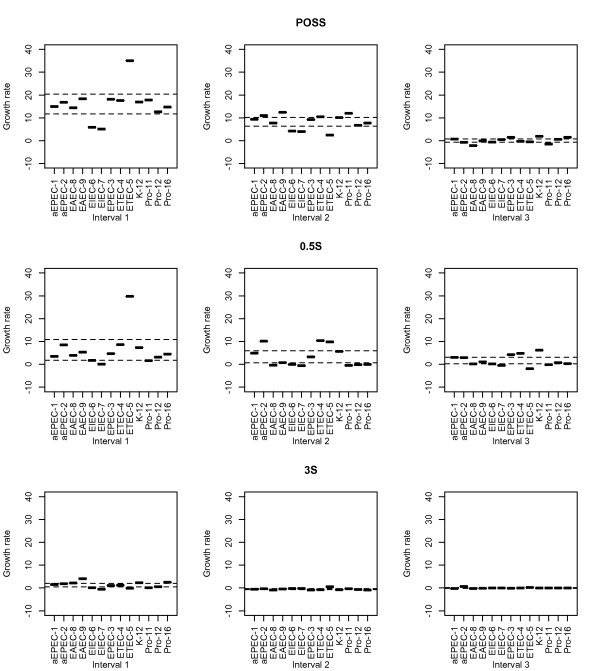
**Comparison of growth rate coefficients in Syncase broth.** The diagrams depict growth rate coefficients as estimated with the spline regression model between the different strains in interval 1, 2 and 3 cultured in Syncase broth with 0, 0.5 and 3 mg/ml (designated POSS, 05 S, 3 S, respectively) lactoferrin added. The horizontal axes represent the different strains, while the growth rate, measured as change of OD per interval x 1000, is given on the vertical axes. Dashed horizontal lines represent 95% confidence intervals for each interval in each trial (*i.e.* POSS, 05 S, 3 S). Growth rates, here represented as small bars, above the 95% confidence intervals were regarded as significantly higher compared to the growth rates within or below the dashed lines. Similarly, growth rates below the 95% confidence intervals were regarded as significantly lower than the growth rates within or above the dashed lines.

A growth curve model was fitted using spline regression for a subset of *E. coli* strains (Table 1). The regression model was developed using linear b-splines with two knots producing three intervals (Figure 1). These three intervals will be referred to as intervals 1, 2 and 3. We used linear splines since we wanted to compare the strains‘growth rates at different intervals (more precisely, change in absorbance (OD) per interval). Since we wanted to compare growth of many different strains over identical time intervals we opted for fixed intervals instead of the traditional lag-, log- (exponential) and stationary- phases, which vary in duration from strain to strain. The linear spline regression model was used to estimate the growth rates of all strains at all intervals in two different broths with different concentrates of lactoferrin added. In other words, the strain comparisons were based on growth rates for each interval from each concentration taken from both broths. All growth rate coefficients from each strain and all intervals were subsequently compared using confidence intervals based on t-distributions. The results are shown in Figures 2 and 3. Growth rates outside the 95% confidence interval were considered as significantly different from the growth rates within.

**Table 1:** Bacterial strains used in the study

Name	Original number	Species	Obtained from
aEPEC-1	NIPH-11060747	atypical enteropathogenic *E. coli* (aEPEC)	Norwegian Public Health Institute (NIPH)
aEPEC-2	NIPH-11061825	aEPEC	NIPH
EPEC-3	NIPH-10208355	typical enteropathogenic *E. coli* (tEPEC)	NIPH
ETEC-4	NIPH-11080893	enterotoxigenic *E. coli* (ETEC)	NIPH
ETEC-5	NIPH-11082604	ETEC	NIPH
EIEC-6	NIPH-11042673	enteroinvasive *E. coli* (EIEC)	NIPH
EIEC-7	NIPH-11042233	EIEC	NIPH
EAEC-8	NIPH-11080035	enteroaggregative *E. coli* (EAEC)	NIPH
EAEC-9	NIPH-11082679	EAEC	NIPH
Pro-11	S2G3/10	probiotic *E. coli*	Symbio Herborn Group
Pro-12	S2G1/2	probiotic *E. coli*	Symbio Herborn Group
Pro-16	Nissle 1917	probiotic *E. coli*	Technische Universität Dresden, Germany
K-12	ATCC47076, MG1655	commensal *E. coli*	ATCC

### Growth in LB and Syncase broth

**Figure 4:**
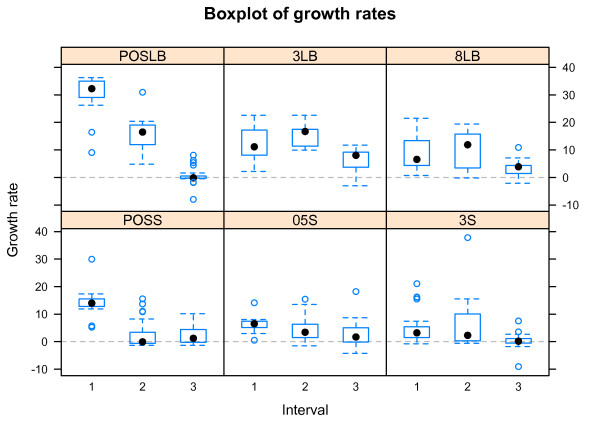
**Boxplots of growth rate coefficients.** Boxplot of growth rate coefficients of all strains as estimated with the spline regression model for interval 1, 2 and 3 and all trials. The title of each box designates the concentration of lactoferrin added (POSLB, 3LB, 8LB, designates 0, 3 and 8 mg/ml, respectively, for strains cultured in LB broth, while POSS, 05 S and 3 S, designates 0, 0.5, 3 mg/ml, respectively, for strains cultured in Syncase broth). The horizontal axes represent interval, while the vertical axes represents the estimated growth rate.

All strains were grown in both LB and Syncase broth, and the empirical growth curves are shown in Figure 1 together with the modeled curves. For all strains growth rates were calculated for each time interval 1 to 3, and subsequently compared according to the guidelines given above (Figures 2 and 3 for LB and Syncase broth, respectively). Growth rates from each interval and all strains are shown in Figure 4. Differences in growth rates between LB and Syncase broth can be observed from the box plot.

The growth rates were higher, and varied more when the strains were cultured in LB broth (*F = 1.9*, *p < 0.001*). This was especially pronounced in interval 1 for the positive controls (POSS/POSLB, *p < 0.005*) where the exponential phase occurred. Growth was, in general, lower in Syncase broth in interval 1 for all concentrates of lactoferrin (3 or 0.5 mg/ml *p < 0.05*, 8 or 3 mg/ml *p < 0.005*, for LB and Syncase, respectively). The growth rates for the positive controls in interval 2 (containing mid exponential phase or start log phase, see Figure 1) were also significantly lower in Syncase than in LB broth (POSS/POSLB, *p < 0.001*). The growth rates for the positive controls in LB broth taken from interval 2 were lower than the ones taken from interval 1. This was probably due to the fact that many strains had already reached their peak of the exponential phase in interval 1.

### Lactoferrin mediated growth effects between the different strains

Figures 2 and 3 show the variation in growth rates for all strains when different concentrations of lactoferrin were added. Thus, growth rates outside the 95% confidence interval were considered significantly different to those within the confidence interval.

The growth rate of most strains declined when increasing concentrations of lactoferrin were added. Concentrations of 3 and 8 mg/ml lactoferrin added to LB broth did not inhibit growth completely in all strains, but growth rates declined considerably, especially when 8 mg/ml was added.

Some strains showed a significantly lower growth rate in LB broth with lactoferrin added compared with other strains. This is particularly visible for the EIEC-6, EIEC-7 and Pro-11 strains. As shown in Figure 1, EIEC-6 exhibited poor growth in LB broth regardless whether 3 or 8 mg/ml lactoferrin was added. The strains aEPEC-2, EAEC-8, EAEC-9 and Pro-16 all grew faster than the other strains in LB broth when both 3 and 8 mg/ml lactoferrin was added. The growth rates of these strains were predominantly higher in interval 1, and continued over to interval 2 when 8 mg/ml was added. In general, growth rates were lower in Syncase compared to LB broth (Figure 4). When the lowest concentration (0.5 mg/ml) of lactoferrin was added to Syncase broth limited growth was observed for the following strains: EIEC-6, EIEC-7, EAEC-8, EAEC-9, Pro-11, Pro-12 and Pro-16 (Figure 1 and 3). The growth rates of EIEC-6, EIEC-7 and Pro-11 were lower in interval 1 when both 0.5 mg/ml and 3 mg/ml lactoferrin were added to Syncase. The growth rate of ETEC-5 was exceptionally high both when 0 (positive control) and 0.5 mg/ml lactoferrin was added to Syncase broth. Hence, 0.5 mg/ml lactoferrin appeared to have only minor growth inhibiting effects on the ETEC-5 strain in interval 2 for Syncase broth compared to the other strains tested. In general, all strains, including ETEC-5, exhibited relatively poor growth in Syncase broth when 3 mg/ml lactoferrin was added.

### GAM based growth model

**Figure 5:**
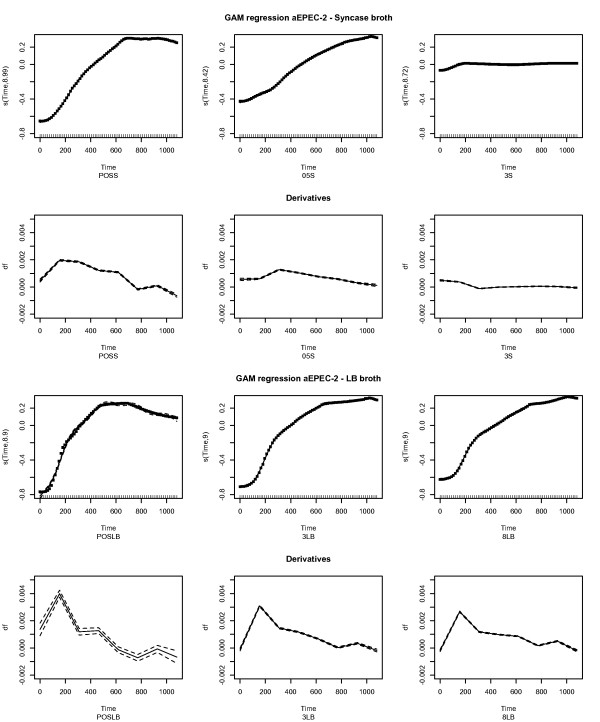
**GAM modeling of the aEPEC-2 strain.** The Figure shows a GAM regression model and the respective derivatives of the growth curves of the aEPEC-2 strain. For the GAM regression model the y-axis designates the scaled OD scores produced with splines with the given degrees of freedom. The x-axis depicts time in minutes. The small black dots on the GAM curves represent the residuals and hence give an impression of the goodness of fit of the GAM models. The figures denoting the respective derivatives of the GAM modeled growth curves show the derivative function of the respective growth curve on the y-axis with corresponding minutes of the x-axis. It can be seen that while growth is generally slow for Syncase broth with lactoferrin added, growth is increasing more abrupt in LB broth before it declines.

Figure 5 shows a more detailed GAM regression model of the pathogenic aEPEC-2 strain together with computed derivatives. It can be observed that for LB broth growth increased substantially just short of the first 200 minutes, as compared to growth in Syncase broth, before it slowed down and declined. The derivatives varied more in LB broth than in Syncase broth.

## Discussion

### The effects of lactoferrin as a growth inhibitor on a set of E. coli strains

Growth differences seemed to be smaller among the strains when cultured in Syncase broth as compared to LB broth. This was especially pronounced when the highest concentrations of lactoferrin were added to the respective broth, *i.e.* 3 mg/ml and 8 mg/ml for Syncase and LB broths, respectively.

The enteroinvasive *E. coli* strains, EIEC-6 and EIEC-7, grew significantly slower than the other strains, especially in LB broth for interval 1, but also for the positive controls of both broths. The slow growth could be due to their invasiveness to eukaryotic cells. The atypical enteropathogenic *E. coli,* aEPEC-2, grew on average faster in both growth media than the other strains and appeared to be more resistant to the growth inhibiting effects of lactoferrin than the other strains. This was particularly noticeable in interval 1 and 2. Even when 8 mg/ml lactoferrin was added to LB broth the growth of the aEPEC-2 strain continued to thrive. In Syncase broth with 3 mg/ml lactoferrin no growth were observed.

When maximum concentrations of lactoferrin were added to the respective broth (8 mg/ml and 3 mg/ml, for LB and Syncase, respectively) larger growth variances were observed among the strains in LB broth than in Syncase broth. However, the addition of 3 mg/ml lactoferrin in Syncase broth eliminated or reduced growth in all strains to a minimum. LB is a rich nutrient medium, while Syncase is a defined growth medium, low in iron content. Previous studies have reported that lactoferrin could deprive iron from the bacteria [4,5] and this might explain why bacterial growth is more inhibited in Syncase broth than in LB broth although the same concentrations of lactoferrin were added. Decreased virulence was also a reported mechanism, but this is probably not prominent in our study since this is most likely associated with adhesion to epithelial cells and not bacterial growth as such.

The enterotoxigenic *E. coli*, ETEC-5, exhibited more anomalous growth trends than the other strains when cultured in Syncase broth. The ETEC-5 strain exhibited fast bursts of growth before correspondingly abrupt phases of growth inhibition. Interestingly, such growth tendencies were not observed in the nutrient rich LB broth. In a previous study several ETEC strains were tested for lactoferrin inhibition and a large strain variation was reported [14]. The effect of lactoferrin was shown to be dependent on strain, dose, iron saturation of lactoferrin, and bacterial inoculums.

Most studies reporting antibacterial effect of lactoferrin have used one or only a few *E. coli* strains and, predominantly, one growth medium in the experiments [5,15-17]. Consequently, MIC values and growth effects may be hard to compare between distinct studies. Not surprisingly, growth media has been shown to have a large impact on growth rate both with and without lactoferrin. Using the proposed procedure described above we could compare many strains on different broths exposed to many measurable factors in a fast and reliable way up to statistical accuracy. Our study clearly demonstrated a large variation between *E. coli* strains. Hence, *E. coli* cannot be seen as one uniform group with regard to the effect of lactoferrin. Taken into consideration the low number of strains from each pathogroup, these results do not indicate clear differences between pathogroups or pathogenic versus non-pathogenic *E. coli.* One exception could be EIEC, which in general, seems to grow with a considerably lower rate than the other pathogroups. However, Dionysius *et al.*[14] demonstrated that even the ETEC pathogroup could not be seen as one uniform group when it came to lactoferrin resistance.

### Interpretation of GAMs and spline regression models

One of the advantages of using regression analysis when modeling bacterial growth is that predictor variables can be incorporated into the model. Such predictor variables can contain information about the growth media, environment, temperature, etc. and may in turn shape the growth curves. In other words, external factors can be tested for effects on growth.

Generalized additive models (GAM) and spline regression models add smaller basis splines together to make a larger spline that fits as closely as possible to the empirical data. Hence, the coefficients in the resulting spline regression model represent the degree of scaling applied to each basis spline [18]. This makes it difficult to interpret the resulting regression coefficients in terms of the observed data since they are calculated for several smaller splines that are scaled between each knot making up the curved line that is being modeled. In other words, the calculated spline coefficients are in fact scaling coefficients that do not necessarily reflect the bacterial growth rates. This is also true for linear b-splines (splines of polynomial degree = 1), as these splines consist of multiple lines joined together as curves. Thus, each basis spline, linear or non-linear, may have many derivatives for each knot since many lines/curves make up a basis spline. Luckily, statistical programs are available that can calculate the slope of the joint line segments, as opposed to the scaling coefficients, so that time consuming multiple linear regressions will not have to be performed. In the present work the bacterial growth curves were compared using a standard linear spline regression model of which the slope of the growth estimates could be obtained. The slopes were subsequently compared using t-distributions, which allowed us to check whether the growth rates, *i.e.* the calculated slopes of the different growth curves, increased or decreased when compared between the different strains. Although the t-distribution estimated the confidence intervals for the data used here adequately, other distributions or bootstrap re-sampling can be used instead, should the data demand it. This procedure makes it both easy and fast to compare many bacterial growth curves with statistical certainty.

Figure 5 shows that GAMs are capable of modeling bacterial growth curves with considerable precision. In addition, derivatives can be computed for all terms in the regression model implying that a detailed picture of growth difference trends can be obtained. This allows for a more thorough examination of how growth changes with time.

Although a confidence interval can be obtained, the GAM and spline regression models close fitting capabilities can also make it difficult to compare the different strains. Indeed, small perturbations may result in similar growth curves being regarded as different by the GAM and spline regression models. Therefore, GAMs may be more appropriate in a bacterial growth curve modeling effort, but not as a method to assess growth differences up to statistical certainty.

Although the present study is concerned with assessing growth differences between a set of *E. coli* strains the term growth differences is, strictly speaking, somewhat misleading. We described growth as measured using optical density (absorbance), but it has been shown that the OD measure is not completely congruent to viable counts (CFU/ml) [19]. We do not, however, believe that this is a problem here since we are only interested in assessing growth difference, up to statistical certainty, between the strains. In addition, due to the conservativeness of the procedure here described, only large differences in growth are detected. This is an intended property of the procedure so that it is more likely that differences found are due to biological differences and not to natural random variations in growth. A limitation of the proposed model is therefore that strains exhibiting small growth differences, instigated by biological differences, may not be detected. A weakness of the OD based method is that antimicrobials can affect the maximum density reached by the cells meaning that microbial growth can occur without being detected using OD. This is nevertheless a weakness that has no bearing on the statistical method proposed for comparison, which can also be used with viable counts data.

## Conclusions

We found that the spline regression models were easy to set up and well suited for comparing differences in bacterial growth. The regression models helped elucidate growth differences, as measured using absorbance, in several *E. coli* strains cultured in Syncase and LB broth subjected to different concentrations of lactoferrin.

It was found that the *E. coli* strains grew faster in LB broth than in Syncase broth regardless of the concentration of lactoferrin added. In general, the enteroinvasive strains*,* EIEC-6 and EIEC-7 were found to be the slowest growers, while the aEPEC-2 was the fastest. The enterotoxigenic *E. coli*, ETEC-5, exhibited anomalous growth trends compared to the other strains when cultured in Syncase. In LB broth, however, ETEC-5 showed no strange growth properties. The atypical enteropathogenic *E. coli,* aEPEC-2, was overall more resistant to lactoferrin than the other strains. In both media growth was substantially faster in interval 1, which was faster than in interval 2, which, in turn, was faster than in interval 3, as expected for normal bacterial growth curves. In general, no distinct growth differences were detected between the different pathogroups with the possible exception of the EIEC group.

The growth of the aEPEC-2 strain was also modeled using a more advanced GAM model to elucidate growth differences for different concentrations of lactoferrin added. The GAM modeled the growth curves with an impressive degree of precision. In addition, the ability to obtain derivatives with confidence intervals, implies that GAMs can reveal growth trends of the modeled strain in more detail. The close fit to the empirical data will however not make it easier to compare growth between the strains. Although there are methods that can facilitate such comparisons they are quite complicated [20,21]. Nevertheless, the GAMs, and the corresponding derivatives, allows for a more detailed analyses of how bacterial growth changes over time.

## Methods

### Bacterial strains and growth conditions

Thirteen *E. coli* strains, pathogenic and non-pathogenic, were included in the study (Table 1). The strains were grown in Luria Bertoni broth (LB) (Sigma-Aldrich, Missouri, USA) overnight at 37 °C, then diluted 1:100 in LB (rich nutrient broth) or Syncase broth (low iron content, contains less than 0.5 μg of Fe^3+^ per ml) [22]. From each of the 1:100 dilutions 250 μl of the strains were distributed to each well in the 96-well Nunclon^TM^Surface microtiter plates (Nunc AS, Roskilde, Denmark). The inoculum concentration was 1x10^5^-5x10^6^ colony forming units. Different concentrations of bovine lactoferrin (approximately 15% iron-saturated, DMW International, Veghel, Netherlands) were added to the wells and incubated at 37 °C in a microtiter plate reader (Sunrise Remote Control, Tecan, Salzburg, Austria). To measure bacterial growth, optical density was monitored at wavelength 620 nm every 15 min for 18 hours (1080 minutes). The start inoculum and the negative control, growth medium without bacteria, had similar OD detection level (≥0.1). The modeled growth curves were based on empirical triplicate measures of bacterial growth from each sample. Positive controls were 250 μl of all *E. coli* isolates grown in both LB and Syncase broth with added water (50 μl) in order to obtain identical volumes for all samples. All *E. coli* strains were cultured using 3 and 8 mg/ml of lactoferrin (Lf) when grown in LB broth, in addition to the positive control without lactoferrin. In Syncase broth, a defined growth media with low iron content, 0.5, and 3 mg/ml of lactoferrin were added, in addition to the positive control without lactoferrin. 8 mg/ml lactoferrin was also added to Syncase broth, but since most strains were totally inhibited by 3 mg/ml these results were not included in this study.

### Software and packages used for analyses

Statistical analyses were carried out using the statistical programs R, http://www.r-project.org/, and STATA (StataCorp. 2009. Stata Statistical Software: Release 11. College Station, TX: StataCorp LP.). The “mgcv” and “spline” packages were used to generate the GAM and spline regression-based models, respectively, in R. And the regression models were plotted using the “lattice” package, also in R. The linear growth rates were computed with STATA using the *mkspline* command. Residual distributions were verified to be approximately normal for all parametric models used.

### The regression models

The mathematical/statistical modeling was based on linear basis splines (b-splines) modeling “time” with 2 knots resulting in three growth rate estimates, one for each interval (Figure 1):

(1) $$y_{\mathrm{ijkl}}=b_{0}+b_{1}X+b_{2}x_{i}\cdot x_{j}\cdot f\left(x_{\mathrm{ijkl}}\right)+x_{\mathrm{ijkl}}\cdot {Ζ}_{k}+\epsilon_{\mathrm{ijkl}}$$

Absorbance (OD) *y*_*ijkl*_ represents the outcome where the subscripts *i,j,k* and *l* represents concentrate, strain, replicate and time, respectively. Concentrate *x*_*i*_, strain *x*_*j*_ and the linear b-spline modeling time *f(x*_*ijkl*_*)* were also included in a three-way interaction designated by (*). The matrix **X** consists of all combinations of predictors (*x*_*i*_*, x*_*j*_*, f(x*_*ijkl*_*), x*_*i*_*·x*_*j,*_*x*_*i*_*·f(x*_*ijkl*_*), x*_*j*_*·f(x*_*ijkl*_*)*) from the previously described interaction term and **b**_**1**_ is a vector containing the corresponding parameter estimates. **b**_**2**_ is a vector containing the parameter estimates for the three-way interaction term *x*_*i*_*·x*_*j*_*·f(x*_*ijkl*_*)* and *b*_*0*_ is the intercept. The linear slopes are estimated by the linear spline function *f .x*_*ijkl*_*·Ζ*_*k*_ is the random effect designating replicate *k* with respect to time *l*, while *ϵ*_*ijkl*_ describes the normally distributed errors. Figure 1 shows the fitted values (red lines) against the OD-based growth curves (blue lines) from the above-described regression model. Since the “spline” package in R produces scaling coefficients that can be difficult to interpret, we re-run the above mentioned regression model in STATA using the *mkspline* function, which gives linear coefficient estimates for each slope. Hence, to obtain linear growth estimates, regression models were fitted for all strains using the *mkspline* function in STATA, which provides the slope of each linear growth estimate making up the modeled curve. Both the *bs* function in R and the *mkspline* function in STATA were applied to make a linear spline with three separate, equally spaced lines joined together with two knots. These three lines represent intervals 1, 2 and 3 (See Figure 1).

For each growth curve obtained from the above described regression model, the growth rate was calculated as the slope of each line fitted between the knots of every growth curve (Figure 1). That is, the growth rate (alternatively slope) was calculated for the curve in each of the three intervals. The slope of these line segments may be considered as the derivative of each spline between each knot for every growth curve and thus representing growth rate. We refer to these slopes (or derivatives), change in OD with respect to time, as growth rates. The growth rates for all strains were compared using a t-distribution for each concentration of lactoferrin added to LB or Syncase broth. Growth rates outside the 95% prediction interval were considered as significantly different to those inside (Figures 2 and 3).

For the aEPEC-2 strain GAM regression models were additionally fitted using the “mgcv” package in R (Figure 5) for each concentration in both Syncase and LB broth using the gam command with respect to time:

(2) $$y_{\mathrm{ij}}=b_{0}+s\left(x_{\mathrm{ij}}\right)+{Ζ}_{\mathrm{ij}}+\xi_{\mathrm{ij}}$$

*y*_*ij*_ designates the absorbance (outcome) with respect to replicate *i* and time *j*. *b*_*0*_ is the intercept, while *s* represents the spline-based smoother function (defaults to thin plate regression splines) with respect to time *x*_*ij*_. *Ζ*_*ij*_ *= x*_*ij*_*·ξ*_*i*_ is the random effect of each triplicate *i* with respect to time *j*.

The corresponding derivatives were computed for each growth curve using the instructions given by the documentation of the predict.gam command with type=”lpmatrix”.

A standard F test (equality of variance test) was used to compare slope variances between LB and Syncase broth. The comparisons of the strains’ growth rates from each broth for each interval were carried out using standard linear regression with broth as the predictor variable and growth rate as outcome for each interval and corresponding concentration of lactoferrin added.

## Misc

Camilla Sekse and Jon Bohlin contributed equally

## Authors’ contributions

JB wrote the manuscript, suggested statistical procedures and performed statistical analyses. CS carried out the lab experiments, performed biological analyses and wrote the manuscript. ES contributed to the modeling and statistical analyses. GEV suggested and initiated the experiment. All authors read and approved the final manuscript.

## Competing interests

The authors declare that they have no competing interests.
